# Supplementary figures and images for: Effect of common maintenance drugs on the risk and severity of COVID-19 in elderly patients
Source: PLoS One. 2022 Apr 18;17(4):e0266922. doi: 10.1371/journal.pone.0266922 (PMC9015134; doi:10.1371/journal.pone.0266922)

S1 Figure. Consort diagram

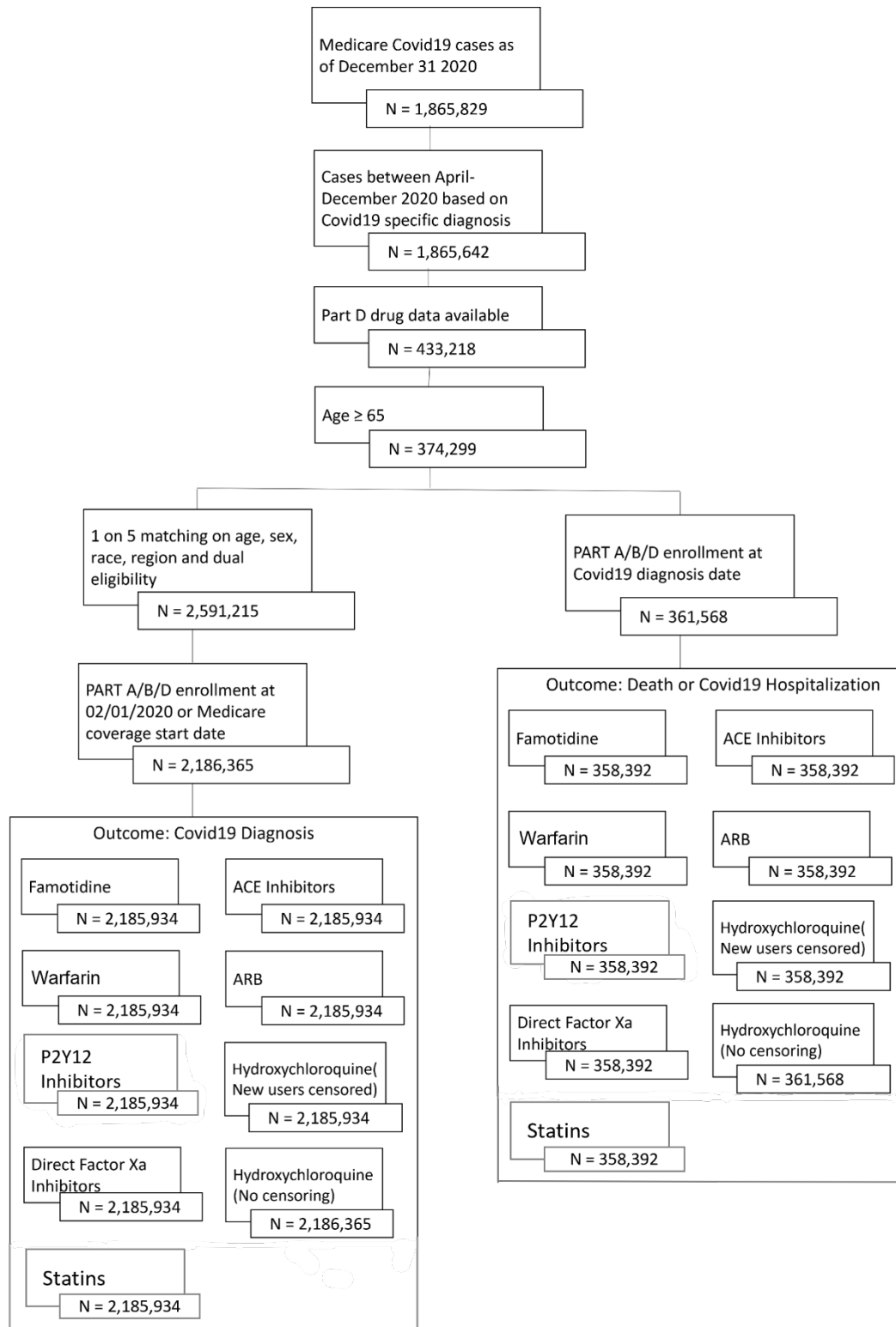

Supplement: S1 Fig — (PDF) [file pone.0266922.s001.pdf]

S2 Figure. Trends of drug usage in 2019 and 2020 (w/o: without; w/: with)

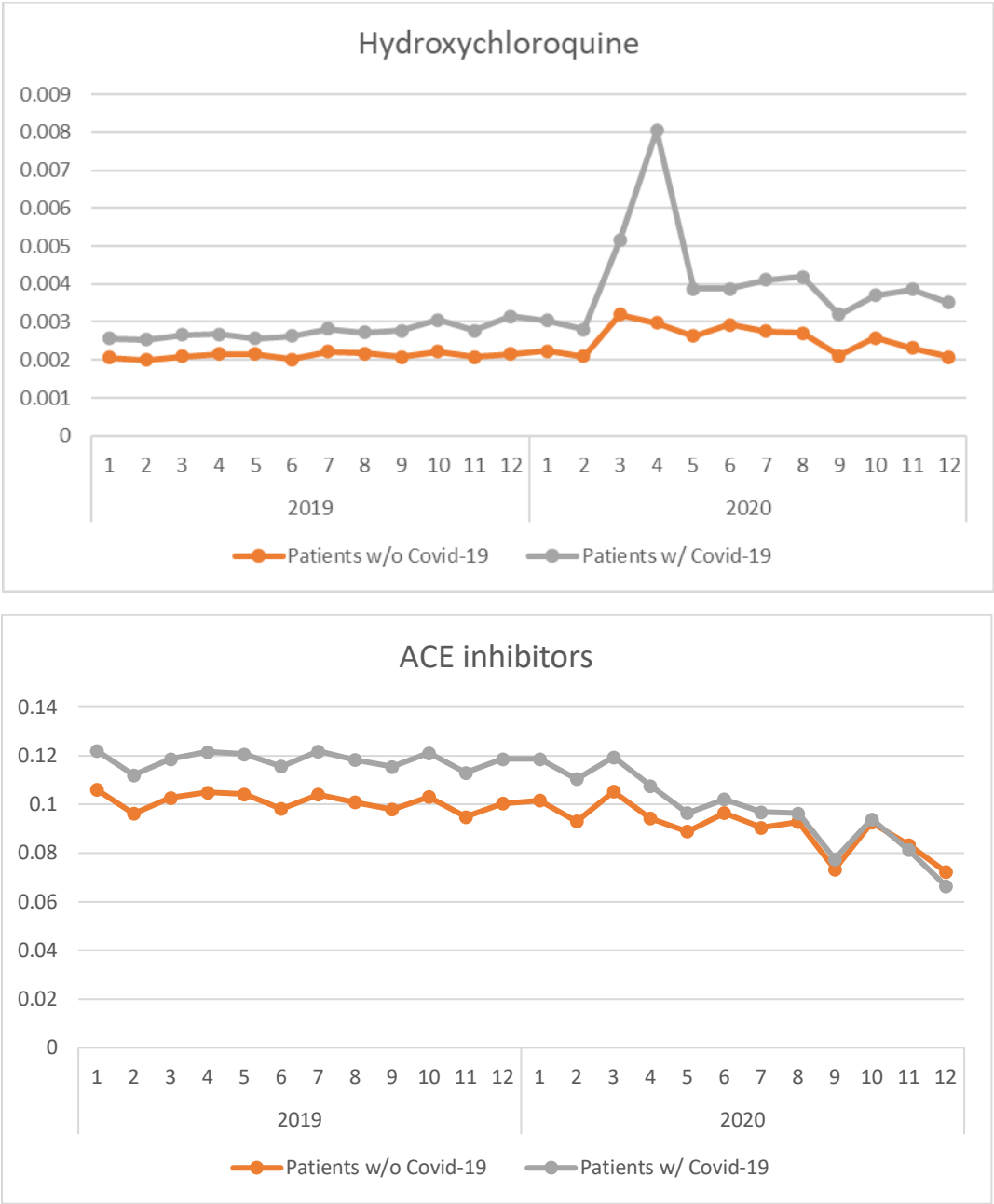

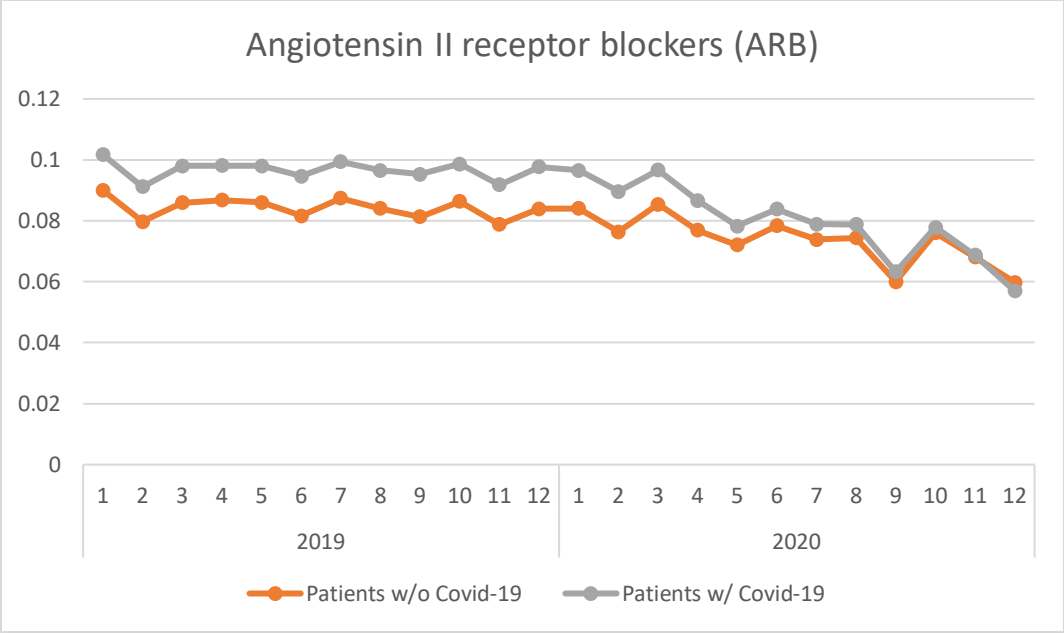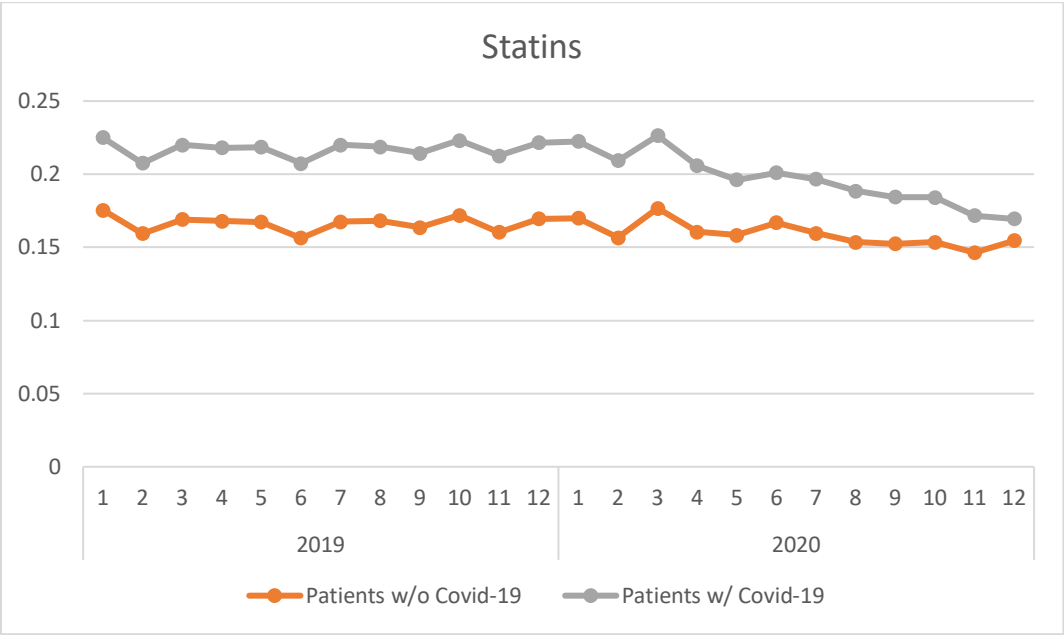

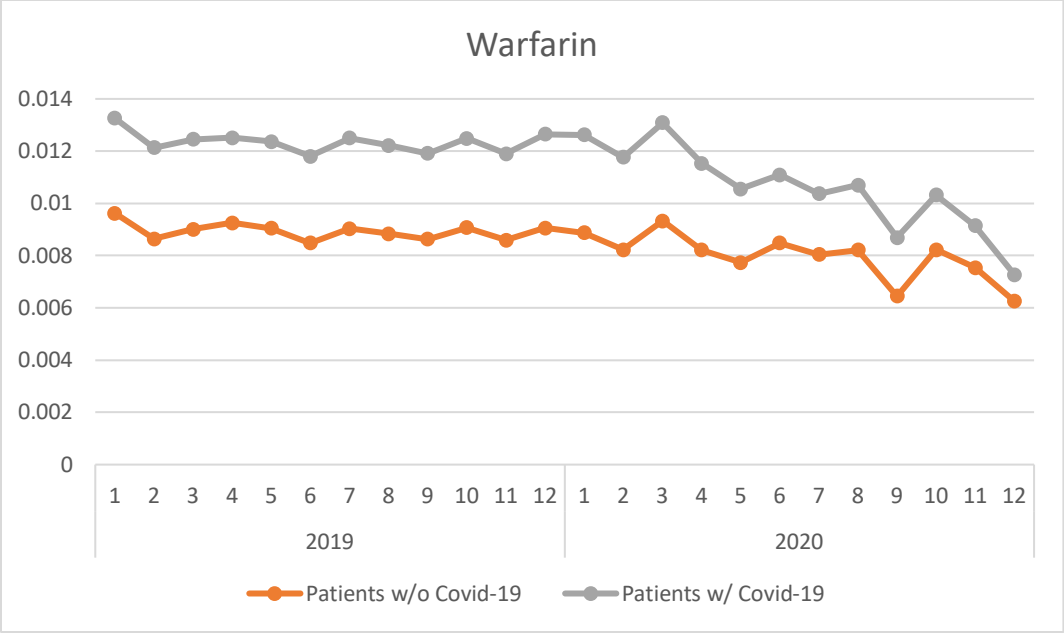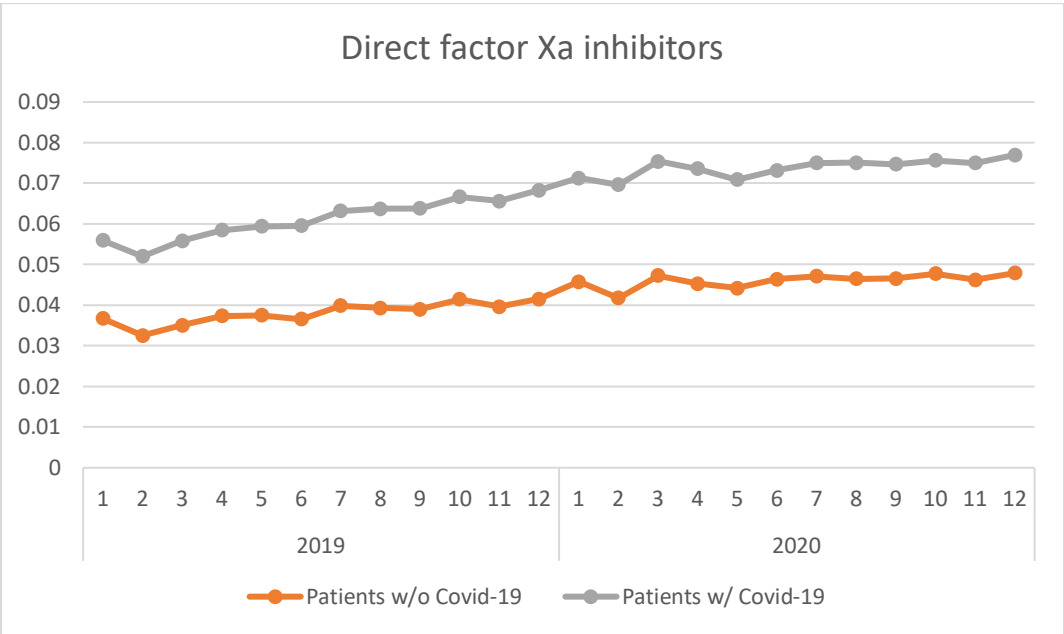

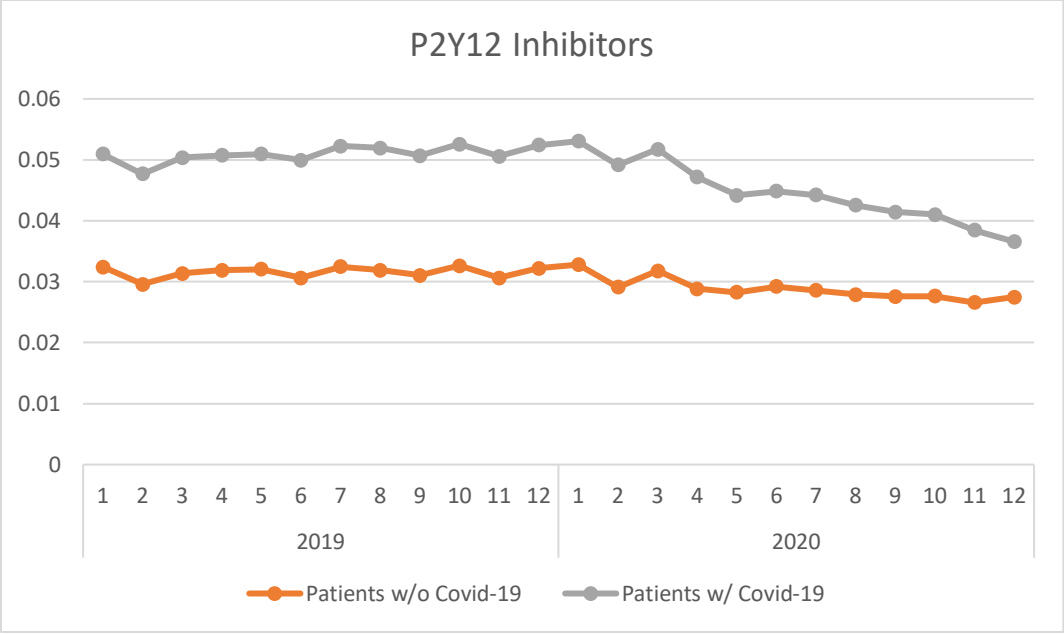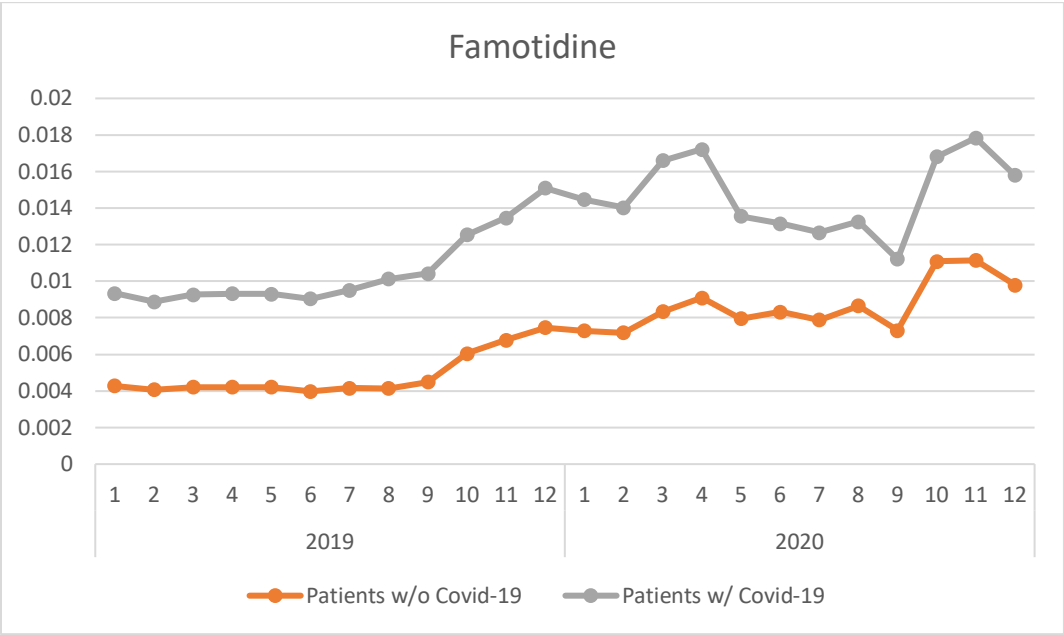

Supplement: S2 Fig — (PDF) [file pone.0266922.s002.pdf]
