# Supplementary material for: Effect of common maintenance drugs on the risk and severity of COVID-19 in elderly patients
Source: PLoS One. 2022 Apr 18;17(4):e0266922. doi: 10.1371/journal.pone.0266922 (PMC9015134; doi:10.1371/journal.pone.0266922)
Supplement: S1 Table — (DOCX) [file pone.0266922.s003.docx]

S1 Table. Drug classes, clinical drugs and usage frequencies among all our study population (ACEI angiotensin-receptor blocker; ARB angiotensin-receptor blocker)

| **Drug class** | **Clinical drug** | **Number of claims** | **% within class** |
| --- | --- | --- | --- |
| ACEI | AMLODIPINE BESYLATE/BENAZEPRIL | 907,955 | 3.03% |
|  | BENAZEPRIL HCL | 1,509,400 | 5.05% |
|  | BENAZEPRIL/HYDROCHLOROTHIAZIDE | 31,077 | 0.10% |
|  | CAPTOPRIL | 41 | 0.00% |
|  | ENALAPRIL MALEATE | 1,610,911 | 5.38% |
|  | ENALAPRIL/HYDROCHLOROTHIAZIDE | 77,437 | 0.26% |
|  | FOSINOPRIL SODIUM | 165,927 | 0.55% |
|  | FOSINOPRIL/HYDROCHLOROTHIAZIDE | 9,167 | 0.03% |
|  | LISINOPRIL | 20,637,915 | 68.98% |
|  | LISINOPRIL/HYDROCHLOROTHIAZIDE | 3,246,361 | 10.85% |
|  | MOEXIPRIL HCL | 8,609 | 0.03% |
|  | MOEXIPRIL/HYDROCHLOROTHIAZIDE | 2 | 0.00% |
|  | PERINDOPRIL ARG/AMLODIPINE BES | 9 | 0.00% |
|  | PERINDOPRIL ERBUMINE | 10,028 | 0.03% |
|  | QUINAPRIL HCL | 519,135 | 1.74% |
|  | QUINAPRIL/HYDROCHLOROTHIAZIDE | 50,329 | 0.17% |
|  | RAMIPRIL | 1,079,221 | 3.61% |
|  | TRANDOLAPRIL | 49,446 | 0.17% |
|  | TRANDOLAPRIL/VERAPAMIL HCL | 3,996 | 0.01% |
|  |  |  |  |
| ARB | AMLODIPINE BES/OLMESARTAN MED | 117,425 | 0.48% |
|  | AMLODIPINE BESYLATE/VALSARTAN | 112,278 | 0.46% |
|  | AMLODIPINE/VALSARTAN/HCTHIAZID | 29,880 | 0.12% |
|  | AZILSARTAN MED/CHLORTHALIDONE | 42,817 | 0.18% |
|  | AZILSARTAN MEDOXOMIL | 54,277 | 0.22% |
|  | CANDESARTAN CILEXETIL | 246,061 | 1.01% |
|  | CANDESARTAN/HYDROCHLOROTHIAZID | 59,711 | 0.25% |
|  | IRBESARTAN | 1,177,484 | 4.84% |
|  | IRBESARTAN/HYDROCHLOROTHIAZIDE | 117,725 | 0.48% |
|  | LOSARTAN POTASSIUM | 15,740,296 | 64.72% |
|  | LOSARTAN/HYDROCHLOROTHIAZIDE | 2,786,140 | 11.46% |
|  | OLMESARTAN MEDOXOMIL | 1,133,630 | 4.66% |
|  | OLMESARTAN MED/AMLODIPINE/HCTZ | 2,988 | 0.01% |
|  | OLMESARTAN/AMLODIPIN/HCTHIAZID | 62,604 | 0.26% |
|  | OLMESARTAN/HYDROCHLOROTHIAZIDE | 451,088 | 1.85% |
|  | TELMISARTAN | 361,087 | 1.48% |
|  | TELMISARTAN/AMLODIPINE | 2,926 | 0.01% |
|  | TELMISARTAN/HYDROCHLOROTHIAZID | 135,302 | 0.56% |
|  | VALSARTAN | 1,020,925 | 4.20% |
|  | VALSARTAN/HYDROCHLOROTHIAZIDE | 667,148 | 2.74% |
|  |  |  |  |
| STATIN | AMLODIPINE/ATORVASTATIN | 46,157 | 0.09% |
|  | ATORVASTATIN CALCIUM | 24,151,835 | 47.86% |
|  | EZETIMIBE/SIMVASTATIN | 118,643 | 0.24% |
|  | LOVASTATIN | 1,901,218 | 3.77% |
|  | PITAVASTATIN CALCIUM | 186,513 | 0.37% |
|  | PITAVASTATIN MAGNESIUM | 347 | 0.00% |
|  | PRAVASTATIN SODIUM | 3,811,097 | 7.55% |
|  | ROSUVASTATIN CALCIUM | 7,556,348 | 14.97% |
|  | SIMVASTATIN | 12,690,686 | 25.15% |
|  |  |  |  |
| HYDROXYCHLOROQUINE | CHLOROQUINE PHOSPHATE | 2,678 | 0.29% |
|  | HYDROXYCHLOROQUINE SULFATE | 916,812 | 99.70% |
|  | PRIMAQUINE PHOSPHATE | 40 | 0.00% |
|  |  |  |  |
| FAMOTIDINE | FAMOTIDINE | 2,146,195 | 99.41% |
|  | FAMOTIDINE/CA CARB/MAG HYDROX | 70 | 0.00% |
|  | FAMOTIDINE/PF | 2,446 | 0.11% |
|  | IBUPROFEN/FAMOTIDINE | 10,178 | 0.47% |
|  |  |  |  |
| WARFARIN | WARFARIN SODIUM | 3,041,385 | 100.00% |
|  |  |  |  |
| P2Y12 INHIBITOR | CLOPIDOGREL BISULFATE | 7,925,920 | 91.66% |
|  | PRASUGREL HCL | 134,340 | 1.55% |
|  | TICAGRELOR | 587,083 | 6.79% |
|  |  |  |  |
| DIRECT FACTOR Xa INHIBITOR | APIXABAN | 8,411,382 | 68.20% |
|  | BETRIXABAN MALEATE | 6 | 0.00% |
|  | EDOXABAN TOSYLATE | 9,230 | 0.07% |
|  | RIVAROXABAN | 3,913,315 | 31.73% |
