# Supplementary material for: Effect of common maintenance drugs on the risk and severity of COVID-19 in elderly patients
Source: PLoS One. 2022 Apr 18;17(4):e0266922. doi: 10.1371/journal.pone.0266922 (PMC9015134; doi:10.1371/journal.pone.0266922)
Supplement: S2 Table — (DOCX) [file pone.0266922.s004.docx]

Supplementary Table S2. The combination use of anti-hypertensive drugs with statins in Medicare patients for 2019 - 2020 (CCB calcium channel blocker; BB beta blocker; ACEI angiotensin-receptor blocker; ARB angiotensin-receptor blocker)

| **Maintenance drug** | **Overall (%)** | **COVID-19 patients (%)** | **Non-covid patients (%)** |
| --- | --- | --- | --- |
| statins (all) | 1,202,014(100) | 205,905(100.0) | 996,109(100.0) |
| CCB + statins | 453,384(37.7) | 79,813(38.8) | 373,571(37.5) |
| diuretics + statins | 478,305(39.8) | 85,644(41.6) | 392,661(39.4) |
| BB + statins | 531,996(44.3) | 97,055(47.1) | 434,941(43.7) |
| ACE + statins | 421,036(35.0) | 67,267(32.7) | 353,769(35.5) |
| ARB + statins | 344,021(28.6) | 57,466(27.9) | 286,555(28.8) |
