# Supplementary material for: Effect of common maintenance drugs on the risk and severity of COVID-19 in elderly patients
Source: PLoS One. 2022 Apr 18;17(4):e0266922. doi: 10.1371/journal.pone.0266922 (PMC9015134; doi:10.1371/journal.pone.0266922)
Supplement: Attachment — Submitted filename: Response to reviewers comments PLOS ONE.docx [file pone.0266922.s007.docx]

PONE-D-21-31663

Effect of common maintenance drugs on the risk and severity of COVID-19 in elderly patients

Dear PLOS ONE editors,

The authors would like to thank the reviewers for their detailed review and thoughtful comments and suggestions. The following is our specific response to the comments.

Reviewer #1: This is nested case-control study derived from the Medicare claims database, to evaluate the association between various medications and the incidence and severity of COVID. In a 20% random sample of the database, COVID-19 cases were matched 1:5 with non-COVID controls. Analyses adjusted for various factors including comorbidities, geographical region, and insurance status. The authors accounted for indication bias for hydroxychloroquine, by censoring recent initiators of the medication. The authors found that several medications were associated with reduced risk of getting COVID and of severe COVID (hospitalization, death).
Overall this is a very interesting study and the authors have done a nice job presenting the findings. The very large dataset provides sufficient power to both evaluate and account for a large number of variables.

I have several comments:

Throughout the manuscript – but especially in the abstract and the Discussion, the authors need to be very careful to avoid implying causation from the observed associations.
Abstract Conclusion- “…was found to be protective against…” needs to be tempered to describe the association. i.e. “was associated with…”

Response: The verbiage throughout the manuscript has been changed as suggested.

Similarly, in the Discussion, instead of saying “risk declined by 15% or greater” – should say it was “associated with” a lower risk; and instead of “beneficial effects”, would say “beneficial associations”.

Response: The verbiage throughout the manuscript has been changed as suggested.

The choice of which medications to evaluate is not well explained. It does not make sense to me to include clopidogrel but not other agents of the same class. If including clopidogrel, you need to include the other P2Y12 agents: ticagrelor, prasugrel, (and ticlopidine – though less commonly used) – similar to how you look at ACEi’s and ARBs as classes. What is the rationale for only using clopidogrel?

Response: Thanks for the suggestion. We have modified the list of drugs as suggested. Clopidogrel is expanded to P2Y12 inhibitors, which include clopidogrel, prasugrel and ticagrelor, there are no patients on ticlopidine in our population.

Similarly, why only famotidine? Is the anti-viral activity unique to the medication and not the others within this class? If so, this should be stated and referenced, and you need to exclude subjects on the other H2 blockers. If not, should include all H2 blockers as a class.

Response: the suggested beneficial effect of famotidine is based on structural homology analysis [Wu et al] and does not apply to other H2 blockers. This has been clarified in the manuscript.

Apixaban should be included as an anticoagulant as well.

Response: thanks for the suggestion. Apixiban is now included in direct factor Xa inhibitors.

In any observational study, some residual confounding may persist – and this limitation needs to be stated.

Response: residual confounding factors is added as a limitation

In addition, patients who are not on any prescription medications at all may differ from those on medications in important ways besides just health status – even though there are claims present to cause them to be included in the dataset, a subset may have less healthcare access, which could influence the associations seen. It would be of interest to see a sensitivity analysis excluding those who are not on any medications.

Response: we have done the suggested analysis. Overall, among our COVID-19 patients and controls, only 2.5% did not have any prescription at all for the period 2019-2020. Because of the small percentage, we do not think that a separate subset analysis is warranted.

Methods -
The authors state “All cases and control were followed from January 1, 2020 until COVID-19 diagnosis, death, disenrollment from Medicare Parts A/B/D or December 31, 2020, whichever came first” – however cases must have been followed beyond their COVID diagnosis, or else outcomes such as death would not be known…Please specify that this is just the follow-up for the first outcome, risk of acquiring COVID.

Response: clarification added to Methods

Are you capturing all the COVID diagnoses? With a 40% hospitalization rate, it would seem that a lot of COVID cases are not actually being captured. Also, mild cases are probably often undiagnosed and thus not captured. You need a discussion of this in the Limitations section.

Response: Compared with national data from CDC, we could be missing about one-third of COVID-19 cases. It is likely that milder cases would be missed since the patients did not seek medical care. This would also explain the higher COVID-19 hospitalization and mortality rate in our study compared to other studies. This is now added as a limitation

Table 3 and Table 4 are quite interesting – just a suggestions, but if color is allowed, it would be nice to use that instead of bold vs italic, to make the significant findings a bit easier to notice.

Response: results are color-coded as suggested

The Discussion section could benefit from a more thorough description and citing of prior literature.

Response: more references are added

Reviewer #2: This is an interesting and important paper. Its focus is on whether drugs that are often taken by older patients might reduce the occurrence of outcomes of SARS-CoV-2 infection. It is a direct examination of whether these drugs can be considered “repurposed” for COVID-19 care. They show that RAS inhibitors and several anticoagulant preparations can be helpful.

The strengths of the study are its very large size, its use of a single database and its very good statistical methods (time varying propensity scoring). It usefully shows that taking hydroxychloroquine was not helpful, a finding that should lay to rest any lingering hopes that it is. The study also shows that famotidine, a drug hyped as having potential benefits, was not beneficial. The study’s inevitable limitation is that it cannot ascertain how well its subjects were adhering to their prescribed medications in the days and weeks immediately before the diagnosis of SARS-CoV-2 infection was made.

Response: implying drug exposure from prescription records is added as limitation

The study has one gobsmackingly glaring omission. The authors have not included statins among the drugs they studied. Statins are taken considerably more frequently by older persons (>65 years of age) than any of the other drugs they studied. There are approximately 50 individual published reports of statin effects in COVID-19 patients and at least 14 meta-analyses of these reports. Twelve studies report inpatient statin treatment is always associated with reduced COVID-19 mortality.

Why did the authors not study statins? They must explain why they excluded statins from their study. If possible, they could re-examine their database to determine whether statin treatment was associated with any COVID-19 outcomes.

Response: Thanks for the suggestion. Statins have been added to the list of study drugs. Statins are indeed associated with beneficial effects. References are added regarding the effects of statins and the possible mechanisms.

The authors must bear in mind that outpatient documentation of statin treatment is unable to document whether statin treatment was continued after hospital admission. There is a very real risk of a rebound effect following statin withdrawal (see Cubeddu LX et al. Pharmacotherapy 2006; 26:1288-96). Whether this effect follows inpatient withdrawal of any of the drugs included in the authors’ study is unknown; the evidence for withdrawal effects for discontinued ACE inhibitors and ARBs is mixed. However, this effect must be considered a possibility and must be discussed by the authors.

Response: this is added as limitation, specifically mentioning the possible rebound effect mentioned by Cubeddu et al.

The authors should acknowledge that many of the drugs they studied are taken in combination preparations and the drugs with which they are combined might have their own effects on COVID-19 outcomes. For example, ARBs are often taken in combination with calcium channel blockers and CCBs by themselves are said to improve COVID-19 outcomes. A sensitivity analysis that focuses only on the effects of losartan (an ARB taken by 65% of all study subjects) and other ARBs alone might help settle this question.

Response: at the time of the study, we focused on the 8 maintenance drugs/drug classes that received the most attention in relation to COVID-19. There could be other drugs (e.g., calcium channel blockers) that could potentially affect the outcome of COVID-19 infection. But generally, the evidence associated with those drugs tends to be weaker and they were not included in our study. It is true that some of those drugs are frequently taken in combination with some of the study drugs. The possible confounding due to drug combination is added as a limitation.
