# Supplementary material for: Effect of common maintenance drugs on the risk and severity of COVID-19 in elderly patients
Source: PLoS One. 2022 Apr 18;17(4):e0266922. doi: 10.1371/journal.pone.0266922 (PMC9015134; doi:10.1371/journal.pone.0266922)
Supplement: Attachment — Submitted filename: Fung.PONE.resubmission.comments.docx [file pone.0266922.s008.docx]

Page 27, first paragraph, last sentence – “In addition, our study gives justification for the need of clinical trials to study the benefits of initiating ACEI or ARB treatment for patients with COVID-19.”

The authors later acknowledge that they could not ascertain whether patients who were taking these drugs as outpatient were continued on treatment after hospital admission. Given this, the authors could expand on the sentence above and suggest RCTs for many of the drugs (especially statins) they found protective when taken as outpatients.
