# Supplementary material for: Effect of common maintenance drugs on the risk and severity of COVID-19 in elderly patients
Source: PLoS One. 2022 Apr 18;17(4):e0266922. doi: 10.1371/journal.pone.0266922 (PMC9015134; doi:10.1371/journal.pone.0266922)
Supplement: Attachment — Submitted filename: Second response to reviewers comments PLOS ONE 0216.docx [file pone.0266922.s009.docx]

PONE-D-21-31663

Effect of common maintenance drugs on the risk and severity of COVID-19 in elderly patients

Dear PLOS ONE editors,

The authors would like to thank the reviewers for their thoughtful comments and suggestions. The following is our specific response.

Reviewer #1: The manuscript is improved, but some concerns remain:

An additional consideration is that very few individuals use a statin alone, in the absence of ACEI/ARBs (as well as anti-platelet medications). This number may be less than 7% of subjects. One must address the co-occurrence of these medications with care. Previous recent studies of medication use among patients with COVID-19 have shown that over 80% of patients on statins also take anti-hypertensive medication. These co-occurrences could be a substantial source of bias if not carefully addressed. I don’t see that this was addressed at all in statistical analyses or study design.

Response: It is true that statins are often co-prescribed with other maintenance drugs, especially anti-hypertensives. In our Medicare population, statins are taken together with ACEI (in 35% of patients on statins), ARB (28.6%), beta blockers (44.3%) and diuretics (39.8%) (see newly added supplementary table 2). This can be a potential confounder if not properly addressed. In our multiple regression analysis, the use of ACEI, ARB and statins are included in the same unified regression model as time-varying covariates. This ensures that the observed effects of each individual study drug are already adjusted for the co-prescription of other drugs included in the model. A new reference [reference 54] is added to explain the statistical control of confounding effects. It is true that the combined use of statins with other anti-hypertensive (e.g., diuretics, beta blockers) has not been adjusted for. However, up to the start of the study, there is no strong evidence that diuretics, calcium channel blockers or beta blockers significantly affect the susceptibility to and severity of COVID-19. It is therefore unlikely that the observed effects of the study drugs on COVID-19 are caused by these other drugs.

Intro

P14 – the authors state that almost all of the primary studies of these maintenance drugs are small. This is no longer true, especially for ACEi/ARB and statins. There are now studies with thousands and thousands of patients. (For instance, studies from the American Heart Association COVID-19 study teams, among others.)

Response: That sentence in the introduction has been modified to “Most of the early primary studies…have been small…”. In the Discussion, it is now stated that: “Recently published studies do have larger patient numbers. One such study supported by the American Heart Association’s Rapid Response Grant COVID-19 is by An et al. [new reference 50 added] with 4,652 patients on ACEI and 2,546 on ARB. However, this is still lower than the number of patients observed in our study, 97,843 on ACEI and 83,290 on ARB.”

Cause of death not specified – so unclear whether statins, ACE/ARB etc are reducing CVD mortality or COVID mortality. The fact that they often occurred a median of 14 days after a diagnosis could also reflect, in part, increased testing among any hospitalized patient during this time period (i.e. prior to any cardiac procedure or surgery.)

Response: This point is well taken. To elaborate on this issue, the following is added to Discussion: “We used all-cause mortality because of the lack of an accurate cause of death in CMS data. Because of this, it is possible that some reduction of mortality is due to the general protective effect of the study drugs and not related to COVID-19 per se (e.g., statins and hypotensive agents can reduce cardiovascular mortality). However, since the mortality rate was 17% in our COVID-19 patients overall, 29% among those hospitalized, and death occurred within a median of 14 days of COVID-19 diagnosis, it is highly likely that COVID-19 was the main contributor to mortality. Another potential confounding factor is that some patients could be diagnosed with COVID-19 because of more frequent routine testing during an encounter for another reason (e.g., preparation for coronary bypass), which makes COVID-19 an incidental finding rather than the trigger event. However, overall, only 3.2% of our Medicare population were diagnosed with COVID-19, so the chance of a purely incidental COVID-19 diagnosis is small.”

Limited to Medicare population – more access to healthcare, might not apply to other populations.

Response: The sentence is now qualified. “In our study, the group with the lowest income (dual-eligible) was associated with a reduced risk of death. One possible explanation is that this group has better access to healthcare, since patients are eligible to both Medicaid and Medicare. However, this explanation only applies to the Medicaid and Medicare patients and not low income patients in general.”

P21, last 2 paragraphs – grammar needs some attention (i.e. “older age”, not “older patients”, is associated with reduced risk; “current use of”, not “current uses of” hydroxychloroquine). Recommend “female sex” was “associated with” a reduced risk, not “Female experienced a 11% risk reduction” [sic]; and “older age”, not “older patients” were associated with decreased risk. Similar changes on p25 and p26 are recommended.

Response: text modified as suggested, thanks.

Tables 3 & 4 – Please list in the footnote for each Table what covariates these analyses are adjusted for.

Response: A note is added to the title of each table to refer to the other table for covariates that are adjusted for.

The Discussion of why these classes of medications (especially ACEI/ARBS and statins) may be associated with benefits is grossly oversimplified.

Response: Detailed discussion of the possible pharmacologic mechanisms for the benefits of the study drugs is beyond the scope of this paper. We can only highlight the most predominant hypotheses. Newer references [references 45, 48] are added for the possible pharmacologic effects of ACEI/ARB and antithrombotics.

Reviewer #2: Please see my comments to the editor and to the authors. I am uncertain about whether the authors have made their data available to others. See item 4 above.

Response: We’ve submitted new data and modified the Data availability statement as follows: “Concerning data availability, the minimal data set is included in the Supporting information. All data in Supporting information can be used without restriction. This includes the precise values used to build the drug usage trend graphs (S3 Table), and the detailed statistical data obtained in the three Cox regressions (S4 Table), from which the hazard ratios can be derived. As for raw data, CMS does not let us download (or distribute) any patient level data. The data stay on their machine, and we analyze it with software they provide on their machine. The shared detailed statistical data, that we are allowed to take out of their machine, should be sufficient for anyone to verify our results. If researchers wish to access the raw data, they can contact the CMS Virtual Research Data Center. However, data access requires the payment of a fee. Note that the exact set of subjects may not be available even with access to raw data, because our study is based on a randomly selected 20% sample, and CMS will pull a new 20% sample for any new request. However, the results should be almost identical to ours, given the large sample size.”
